# Giant Cell Tumor of Tendon Sheath in Guyon's Canal Causing Ulnar Tunnel Syndrome A Case Report and Review of the Literature

**Published:** 2009-02-02

**Authors:** Ben S. Francisco, Jayant P. Agarwal

**Affiliations:** ^a^School of Medicine, University of Texas Health Science Center at San Antonio; ^b^ Division of Plastic and Reconstructive Surgery, University of Utah Health Science Center

## Abstract

**Objective:** Giant cell tumor of tendon sheath is a rare cause of ulnar tunnel syndrome. We present a case of a 37-year-old woman who presented with decreased sensation and weakness of grip of the right hand. Magnetic resonance imaging indicated the presence of a mass in the hypothenar eminence and showed that the mass was associated with the flexor carpi ulnaris tendon and displacing the ulnar neurovascular bundle. A differential diagnosis included desmoid tumor and sarcoma. **Methods:** Surgical examination showed a mass that was associated with the flexor carpi ulnaris tendon and flexor retinaculum located in the distal portion of Guyon's canal and intertwined with the ulnar nerve and displacing the ulnar artery. The mass was removed and Guyon's canal was released. **Results:** Histological examination indicated a diagnosis of giant cell tumor of tendon sheath (GCTTS). Postoperatively, the patient had fully restored sensory and motor function of the right hand. **Conclusions:** Although GCTTS is the most common solid, soft-tissue lesion of the hand, it is rarely diagnosed properly preoperatively. Therefore, it is imperative to always include GCTTS in the differential diagnosis of any mass of the hand.

Giant cell tumor of tendon sheath (GCTTS) is the most common solid, soft-tissue lesion of the hand.^1^ It most commonly occurs on the tendon sheath and in the joints of the digits.^2^–^4^ It is a benign process that has been known to impinge upon surrounding structures and soft tissue and may erode bony structures as it grows in a confined space.^5^ The literature is scant with descriptions of GCTTS located at the wrist causing ulnar tunnel syndrome.^6^–^11^ Any mass growing within the confined space at Guyon's canal has the potential to cause nerve compression and thus produce symptoms of ulnar tunnel syndrome. Ulnar tunnel syndrome presents as pain in the wrist and the forearm radiating to the ulnar 2 digits, increased pain at night exacerbated by exercise or wrist flexion, numbness, tingling, burning, and prickling in the ulnar 2 digits. It may or may not show weakness and wasting of the intrinsic hand muscles innervated by the ulnar nerve.^12^ We present a case of ulnar tunnel syndrome caused by a localized GCTTS of the hypothenar eminence.

## CASE REPORT

**Figure 1:**
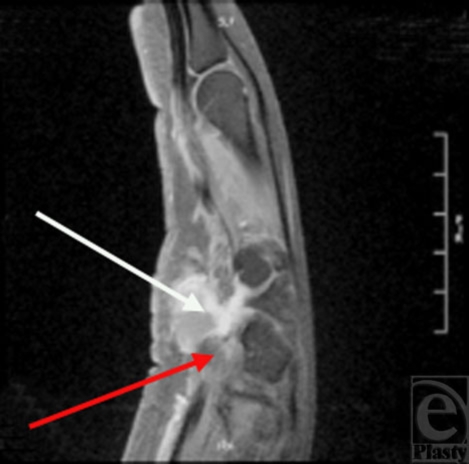
Sagittal T1-weighted magnetic resonance image of the right hand showing a giant cell tumor of tendon sheath involving the ulnar neurovascular bundle at Guyon's canal. The red arrow indicates the ulnar neurovascular bundle. The white arrow indicates the mass.

A 37-year old, right-hand dominant woman who works as a buyer of candy and tobacco for a wholesale distributor presented with decreased sensation and weakness of right-hand grip. Upon examination, a proximal hypothenar fullness was noted. At the time of presentation, the patient indicated that this fullness has waxed and waned for the past 1 year. Over the past 6 months the patient had increased numbness and tingling in the ulnar 2 digits and generalized weakness of her right hand. Ultrasound examination was performed and showed a solid heterogeneous mass of the hypothenar eminence. A follow-up magnetic resonance imaging (MRI) noted a 2.2 × 2.4 × 2-cm bi-lobed mass within the hypothenar musculature, which appeared to be displacing the ulnar artery and ulnar nerve (Fig 1). Based on this MRI a differential diagnosis of desmoid versus sarcoma was given by the radiologist. It was, therefore, determined that incisional or excisional biopsy of the mass would be the most appropriate course of treatment.

**Figure 2:**
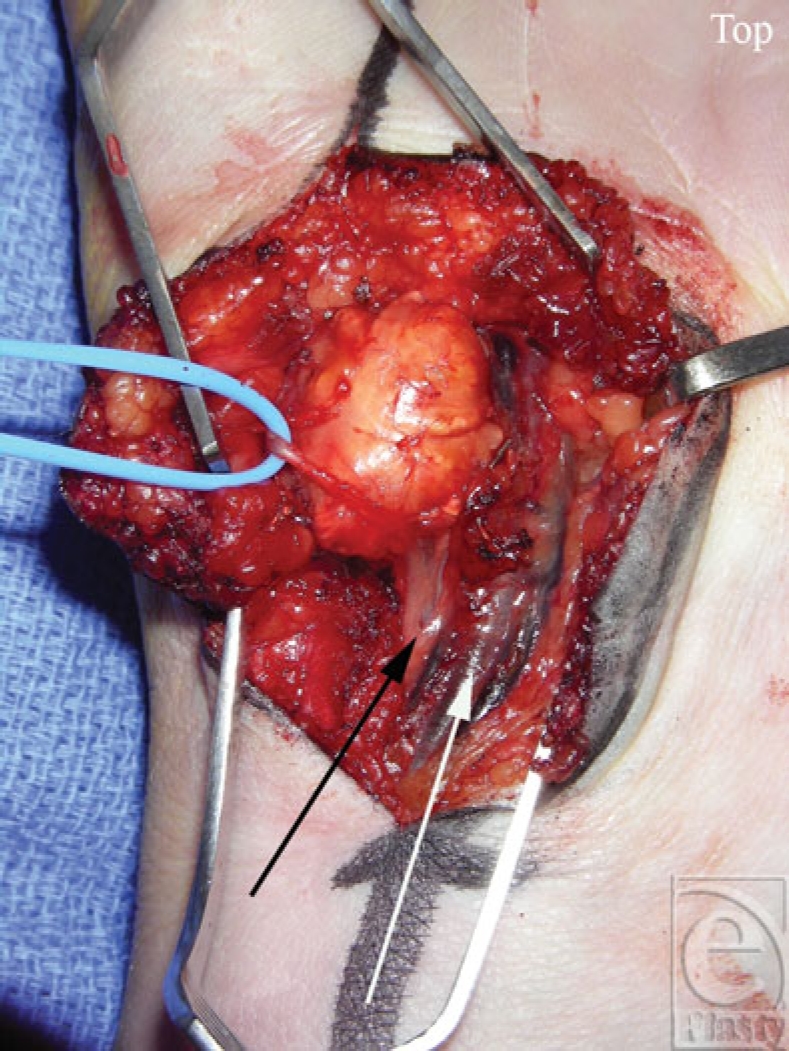
Operative view of the giant cell tumor of tendon sheath entangled in the ulnar nerve. The blue loop is holding the superficial branch of ulnar nerve. The white arrow indicates the ulnar artery. The black arrow indicates the ulnar nerve.

**Figure 3:**
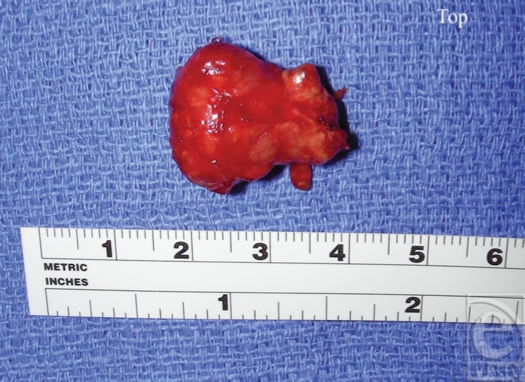
The removed mass.

**Figure 4:**
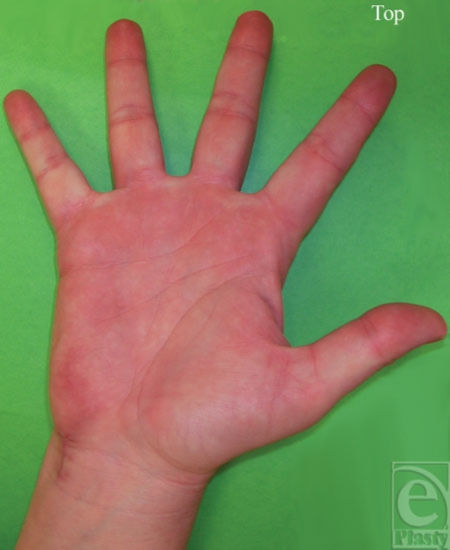
The patient's hand postoperatively.

In the operating room under general anesthesia, a 5-cm Brunner's incision was made directly over Guyon's canal. It extended 3.5 cm distal and 1.5 cm proximal to the wrist crease. The mass was identified and was found to originate at the distal portion of Guyon's canal, extending distally into the hypothenar musculature. The mass was intertwined with the superficial and deep palmar branches of the ulnar nerve proximal to where the deep palmar branch of the ulnar nerve enters the hypothenar tunnel and was impinging upon the ulnar artery (Fig 2). Since the diagnosis was unclear, the hand was not forcefully exsanguinated and care was taken to not disrupt the fascial planes. A small portion of the mass was excised and sent as a frozen section to make the diagnosis. After careful dissection, the lesion was found to be loosely associated with the undersurface of the flexor carpi ulnaris tendon and more associated with the flexor retinaculum. Guyon's canal was released and careful neurolysis was performed. A 2.5-cm, well-circumscribed, bi-lobed mass was removed and sent for pathological evaluation, which indicated a GCTTS (Fig 3). Four months postoperatively, the patient had fully restored sensory and motor function of the right hand (Fig 4).

## DISCUSSION

Jaffe et al^13^ first described GCTTS pathologically in 1941. They postulated that this lesion has a common histological origin with pigmented villonodular synovitis; the major difference being that GCTTS grows outward from the tendon sheath, whereas pigmented villonodular synovitis grows inward from the synovial lining into the joint. There are 3 main cell types that compose GCTTS; these are multinucleated, osteoclast-like giant cells, round or polygonal mononuclear cells, and histiocyte-like cells (foam cells).^14^ All of these cells contain hemosiderin in their cytoplasm.^3^ Gross observation shows lesions that can be yellow, brown, gray-white, and red. Yellowish lesions are laden with lipid, whereas a brown color indicates a predominance of iron. Yellow and brown lesions tend to be associated with GCTTS. The gray-white color indicates the presence of fibrous tissue. The red lesions are typically associated with diffuse pigmented villonodular synovitis.^14^–^16^

While the precise origin of GCTTS is unknown, it has been associated with many different processes including inflammatory, reactive, neoplastic, traumatic, and immune mediated causes. However, based on evidence of DNA flow cytometry^17^, *nm*23 gene expression,^18^ and cytogenetic abnormalities^19^ there is growing evidence that these lesions arise from a neoplastic origin.

Giant cell tumor of tendon sheath can be localized or diffuse. Most often, localized GCCTS is well circumscribed by a thin, fibrous capsule and presents as a small (average, 1.1 cm), slow-growing mass that is firm to the touch, non-tender, and is most often found on tendon sheaths or in the joints of digits.^2^–^4^ Diffuse GCTTS is most commonly associated with the knee and is much less common.^4^ Pigmented villonodular synovitis is identified by a diffusely proliferated synovial membrane with villous projections, may or may not have nodules, and most frequently involves the knee and hip joints. This lesion is usually large (average, 2 cm) and covered by one or more layers of synovial cells.^3^,^4^

The most common benign masses of the hand are ganglion, GCTTS, epidermal inclusion cysts, hemangiomas, fibromas, mucous cysts, and lipomas.^20^–^24^ Giant cell tumor of tendon sheath is the most common solid, soft-tissue lesion of the hand^1^ and is the second most common mass of the hand, second to ganglion.^1^,^20^,^23^ Typically, it is more common in women than men and the mean age of diagnosis is between 30 and 50 years.^1^,^15^

To our knowledge, there have been only 5 case reports on GCTTS located at Guyon's canal in the English literature^6^–^8^,^10^,^11^ and 1 in the non-English literature.^9^ Each case presented with ulnar tunnel syndrome symptoms. In all cases the lesion was impinging upon the ulnar neurovascular bundle in or around Guyon's canal, thereby producing symptoms of ulnar tunnel syndrome. Correct diagnosis was made only upon pathological evaluation.

The sites of ulnar nerve compression within Guyon's canal can be classified into 3 zones. Zone 1 is located distal to the bifurcation of the ulnar nerve into superficial and deep palmar branches. Compression of the ulnar nerve in zone 1 can produce complete sensory and motor deficits, purely sensory or purely motor deficits. Zone 2 surrounds the deep motor branch of ulnar nerve. Compression in zone 2 results in paralysis of the intrinsic muscles and may involve the hypothenar muscles depending upon where the compression occurs in zone 2. Zone 3 surrounds the superficial branch of ulnar nerve. Compression in zone 3 results in purely sensory deficits.^25^,^26^

The most common method for treatment of GCTTS is surgical excision; however, the average recurrence rate is 26.5% (range, 9%–44%) with an average time of recurrence of 2 years 3 months (range, 3 months to 10 years).^27^ Recurrence is common if the lesion is not completely removed. Other factors which increase the rate of recurrence include multiple primary lesions, tumors involving the thumb, and erosion of the bone.^18^,^23^ In addition to excision of the lesion, release of Guyon's canal may be indicated to relieve nerve compression symptoms. The ulnar nerve bifurcates into superficial and deep palmar branches proximal to Guyon's canal. The deep palmar branch of the ulnar nerve continues distally through the hypothenar tunnel, the floor being formed by the piso-hamate ligament and the roof being formed by tendinous origin of a portion of the hypothenar muscles: the adductor digiti minimi, the flexor digiti minimi brevis, and the opponens digiti minimi muscles.^28^ It is important to inspect the deep palmar branch as it travels in the hypothenar tunnel as it may require decompression if the lesion involves the nerve in this structure. Furthermore, once the lesion is removed, the surrounding area should be inspected for satellite lesions and if present these should be removed. If osseous erosion has occurred, removing that part of the cortical shell that has been involved may be indicated to prevent recurrence. Radiation may play a role in the treatment of GCTTS.^14^ However, given the complications of possible hand contracture, radiation is not currently utilized. With regard to more conservative treatment, GCTTS does not resolve on its own.^14^ Therefore, surgical intervention is the only current method of treatment to resolve and relieve symptoms.

Kitagawa et al^29^ state that T1- and T2-weighted MR images of GCTTS located in the hand produce signal intensities between muscle and fat. In addition, they note that the relatively low signal intensity on T2-weighted images of GCTTS is an important feature that differentiates it from soft-tissue sarcoma, which usually has a signal intensity equal to or greater than that of fat on T2-weighted images. Nevertheless, Kittagawa et al speculate that these MRI findings may apply only to the hand. Other MRI studies that have assessed GCTTS included more lesions of the lower extremity, whereas the Kittagawa et al study was composed mainly of GCTTS of the hand (88%). To explain this, they include a finding from Rao and Vigorita^16^ that shows that lesions of the fingers have fewer iron deposits. Less iron serves to shorten the T2-relaxation time due to the magnetic susceptibility effect.^16^ With regard to MRI enhancement, De Beuckeleer et al^30^ report that gadolinium administration enhances GCTTS due to proliferation of capillaries in the stroma. Moreover, Khan et al^31^ state that mild-to-moderate diffuse enhancement is helpful in distinguishing GCTTS from ganglion. Thus, gadolinium enhancement can play an important role in further refining the diagnosis.

## SUMMARY

This case demonstrates that ulnar tunnel syndrome caused by GCTTS is a rare occurrence and that it is often misdiagnosed preoperatively. Since GCTTS is the most common solid, soft-tissue lesion of the hand, it should always be included in the differential diagnosis whenever a mass of the hand is presented. Regarding imaging, MRI with and without enhancement may be useful for diagnosis. Nevertheless, a definitive diagnosis can be provided only by pathologic evaluation. Surgical removal is the standard treatment of GCTTS and it is important that the entire lesion be removed. This is crucial to reduce chances of lesion recurrence. Lastly, release of Guyon's canal and the hypothenar tunnel may be indicated at the time of lesion excision to help relieve nerve compression symptoms.

## References

[1] Karasick D, Karasick S (1992). Giant cell tumor of tendon sheath: spectrum of radiologic findings. Skeletal Radiol.

[2] Rubin BP (2007). Tenosynovial giant cell tumor and pigmented villonodular synovitis: a proposal for unification of these clinically distinct but histologically and genetically identical lesions. Skeletal Radiol.

[3] Ushijima M, Hashimoto H, Tsuneyoshi M, Enjoji M (1986). Giant cell tumor of the tendon sheath (nodular tenosynovitis). A study of 207 cases to compare the large joint group with the common digit group. Cancer.

[4] Ward CM, Lueck NE, Steyers CM (2007). Acute carpal tunnel syndrome caused by diffuse giant cell tumor of tendon sheath: a case report. Iowa Orthop J.

[5] Booth KC, Campbell GS, Chase DR (1995). Giant cell tumor of tendon sheath with intraosseous invasion: a case report. J Hand Surg [Am].

[6] Budny PG, Regan PJ, Roberts AH (1992). Localized nodular synovitis: a rare cause of ulnar nerve compression in Guyon's canal. J Hand Surg [Am].

[7] Hayes CW (1978). Ulnar tunnel syndrome from giant cell tumor of tendon sheath: a case report. J Hand Surg [Am].

[8] Milberg P, Kleinert HE (1980). Giant cell tumor compression of the deep branch of the ulnar nerve. Ann Plast Surg.

[9] Nucci F, Artico M, Antonini G, Millefiorini M, Bastianello S, Bozzao L (1989). Compression of the ulnar nerve in Guyon's canal by a giant cell tumor. Zentralbl Neurochir.

[10] Rafecas JC, Daube JR, Ehman RL (1988). Deep branch ulnar neuropathy due to giant cell tumor: report of a case. Neurology.

[11] Rengachary SS, Arjunan K (1981). Compression of the ulnar nerve in Guyon's Canal by a soft tissue giant cell tumor. Neurosurgery.

[12] Dupont C, Cloutier GE, Prevost Y, Dion MA (1965). Ulnar-tunnel syndrome at the wrist. A report of four cases of ulnar-nerve compression at the wrist. J Bone Joint Surg Am.

[13] Jaffe HL, Lichtenstein L, Sutro CJ (1941). Pigmented villonodular synovitis, bursitis and tenosynovits: A discussion of the synovial and bursal equivalents of the tenosynovial lesion commonly denoted as xanthoma, xanthogranuloma, giant cell tumor or myeloplaxoma of the tendon sheath, with some consideration of this tendon sheath lesion itself. Arch Pathol.

[14] Walsh EF, Mechrefe A, Akelman E, Schiller AL (2005). Giant cell tumor of tendon sheath. Am J Orthop.

[15] Myers BW, Masi AT (1980). Pigmented villonodular synovitis and tenosynovitis: a clinical epidemiologic study of 166 cases and literature review. Medicine (Baltimore).

[16] Rao AS, Vigorita VJ (1984). Pigmented villonodular synovitis (giant-cell tumor of the tendon sheath and synovial membrane). A review of eighty-one cases. J Bone Joint Surg Am.

[17] Abdul-Karim FW, el-Naggar AK, Joyce MJ, Makley JT, Carter JR (1992). Diffuse and localized tenosynovial giant cell tumor and pigmented villonodular synovitis: a clinicopathologic and flow cytometric DNA analysis. Hum Pathol.

[18] Grover R, Grobbelaar AO, Richman PI, Smith PJ (1998). Measurement of invasive potential provides an accurate prognostic marker for giant cell tumour of tendon sheath. J Hand Surg [Br].

[19] Sciot R, Rosai J, Dal Cin P (1999). Analysis of 35 cases of localized and diffuse tenosynovial giant cell tumor: a report from the Chromosomes and Morphology (CHAMP) study group. Mod Pathol.

[20] Bogumill GP, Sullivan DJ, Baker GI (1975). Tumors of the hand. Clin Orthop Relat Res..

[21] Leung PC (1981). Tumours of hand. Hand.

[22] Schultz RJ, Kearns RJ (1983). Tumors in the hand. J Hand Surg [Am].

[23] Smith P (2001). Lister's the Hand.

[24] Sobanko JF, Dagum AB, Davis IC, Kriegel DA (2007). Soft tissue tumors of the hand. 1. Benign. Dermatol Surg.

[25] Gross MS, Gelberman RH (1985). The anatomy of the distal ulnar tunnel. Clin Orthop Relat Res..

[26] Shea JD, McClain EJ (1969). Ulnar-nerve compression syndromes at and below the wrist. J Bone Joint Surg Am.

[27] Reilly KE, Stern PJ, Dale JA (1999). Recurrent giant cell tumors of the tendon sheath. J Hand Surg [Am].

[28] Pecina MM, Krmpotic-Nemanic J, Markiewitz AD (2001). Tunnel Syndromes: Peripheral Nerve Compression Syndromes.

[29] Kitagawa Y, Ito H, Amano Y, Sawaizumi T, Takeuchi T (2003). MR imaging for preoperative diagnosis and assessment of local tumor extent on localized giant cell tumor of tendon sheath. Skeletal Radiol.

[30] De Beuckeleer L, De Schepper A, De Belder F (1997). Magnetic resonance imaging of localized giant cell tumour of the tendon sheath (MRI of localized GCTTS). Eur Radiol.

[31] Khan S, Neumann C, Steinbach L, Harrington K (1995). MRI of giant cell tumor of tendon sheath of the hand: a report of three cases. Eur Radiol..

